# An Evaluation of a Novel Mask in Four Patients with Obstructive Sleep Apnea and Overlap Syndromes

**DOI:** 10.1155/2013/945782

**Published:** 2013-07-18

**Authors:** Alireza Yarahmadi, Nader D. Nader, Gino Zadeii, Jahan Porhomayon

**Affiliations:** ^1^Mercy North-Iowa Neurology and Sleep Laboratory, University of Iowa, Mason City, IA 50401, USA; ^2^VA Western New York Healthcare System, Division of Cardiothoracic Anesthesia and Pain Medicine, Department of Anesthesiology, State University of New York at Buffalo, School of Medicine and Biomedical Sciences, Buffalo, NY 14215, USA; ^3^University of Iowa, Mason City, IA 50401, USA; ^4^VA Western New York Healthcare System, Division of Critical Care and Pain Medicine, Department of Anesthesiology, State University of New York at Buffalo, School of Medicine and Biomedical Sciences, Buffalo, NY 14215, USA

## Abstract

We present four cases of adults with obstructive sleep apnea in whom positive airway pressure therapy alone failed to provide adequate oxygenation. We have previously reported the use of dual mask for ventilatory support of a patient postoperatively (Porhomayon et al., 2013). Here, we report an evaluation of the dual mask in four patients with overlap syndromes. Application of dual mask provided adequate oxygenation with lower continuous positive airway pressure (CPAP)/bilevel positive airway pressure (BIPAP) pressure levels.

## 1. Introduction

Obesity is a worldwide epidemic with numerous studies reporting increases in prevalence of this serious condition [1–3]. Obstructive sleep apnea syndrome (OSAS) is one of the common disorders of breathing and sleep disorder (BSD) in general population [4]. OSAS is also highly prevalent in the obese population. Its prevalence is estimated to be between 2% and 25% in the general population [5]. Since obesity is on the rise, it is logical to assume that the incidence of unrecognized or undiagnosed sleep apnea may be even higher in both medical and surgical population [6]. 

Chronic obstructive pulmonary disease (COPD) and OSAS are an example of overlap syndrome where both diseases affect nearly 10% and 5% of the adult population over 40 years of age, respectively. Both conditions may coexist in about 0.5% of the population. Patients with overlap syndromes may have a greater or more sustained sleep-related oxygen (O_2_) desaturation than do patients with COPD with the same degree of bronchial obstruction. They have an increased risk of developing hypercapnic respiratory insufficiency when compared with OSAS alone. In patients with overlap syndromes, hypoxemia and hypercapnic induced pulmonary hypertension can be observed in the presence of mild to moderate bronchial obstruction. This is different from “usual” COPD because the two syndromes may induce much greater changes in hemodynamic and respiratory as well as neurological functions [7]. The gold standard therapy for OSAS and overlap syndrome remains CPAP or Bi-PAP therapy [8]. In some conditions, CPAP/BIPAP therapy alone fails to provide satisfactory oxygenation. In these situations, O_2_ is often added to CPAP/BIPAP mask. Severe sleep apnea may coexist with other overlap syndromes such as COPD, congestive heart failure (CHF), pulmonary fibrosis, neuromuscular disorders, chronic narcotic use, or central hypoventilation syndrome. They are among conditions that may need additional supplemental O_2_ during the titration process. The aim of this study was to further evaluate the application of dual mask device in four patients in our sleep laboratory before full implementation in a randomized clinical trial.

## 2. Material and Methods

**Figure 1:**
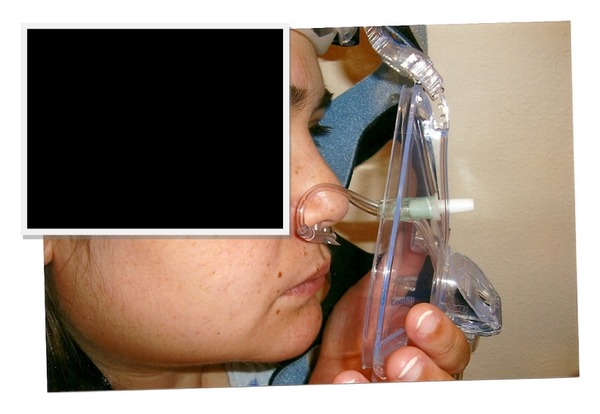
Application of dual mask to patient face.

Dual mask is a system with an adjustable port for connection of nasal cannula (Figure 1). The length of nasal cannula can be adjusted from outside mask by the patient. Mask is connected to a hose that contains both room airs from CPAP/BIPAP and oxygen tube. Nasal cannula dwells within the shell and is connected to an oxygen concentrator. We evaluated the efficacy of the dual mask in the sleep lab in four patients with overlap syndromes. This study was approved by Institutional Review Board (IRB) of the Mercy hospital of North Iowa, and informed consent was obtained from all patients.

## 3. Results

### 3.1. Case  1

This was a 61-year-old female with complaints of excessive fatigue, history of loud snoring, and CHF. She was on acetaminophen with codeine for shoulder pain. Her apnea-hypopnea index (AHI) was 53.5 in her first sleep study. During her second night of polysomnogram (PSG) for CPAP titration, she failed CPAP due to continuous desaturation and frequent apneas and hypopneas. Therefore, she was switched to BIPAP. Despite increasing BIPAP pressure to 22/18 cm H_2_O, she continued to have desaturation until 3 liters of O_2_ was added to her BIPAP. O_2_ saturation stayed in low 90s and high 80s on this setting, but the patient was nervous and unable to tolerate this pressure especially due to leakage from her full-face mask. The patient was scheduled for a second night of CPAP/BIPAP titration. Titration started with dual mask and with 2 liters/min of O_2_ via nasal cannula. At CPAP of 10 cm H_2_O, AHI was 1.2 with good patient tolerance and O_2_ saturation (Tables 1 and 2).

### 3.2. Case  2

This was a 51-year-old male with history of COPD and daytime fatigue. He underwent a split night PSG. His AHI was 35 with minimum desaturation in low 60s. CPAP was applied. Right away, the patient started to have central apneas; therefore, BIPAP was applied. The patient was a major mouth breather, so technician tried every full-face mask with or without chinstrap. None of them kept a good seal due to patient's beard. Low tidal volume and events still noted due to mouth breathing. At BIPAP pressure of 15/11 cm H_2_O, 2 liters of O_2_ was applied, but it did not improve the saturation. Technician was unable to establish a final pressure due to numerous central apneas and desaturation.

Around 0300, dual mask with 2 liters/min of O_2_ was applied and titration started with BIPAP of 8/4 cm H_2_O. At BIPAP pressure of 12/8 cm H_2_O, saturation remained in low 90s with AHI = 1.4 (Tables 3 and 4).

### 3.3. Case  3

This was a 58-year-old male with congestive heart failure, excessive daytime somnolence, and witnessed apnea. His previous PSG was suggestive of mixed obstructive and central apneas with AHI of 44.5. He failed CPAP titration in his first sleep study due to recurrent arousal and desaturation. The patient had 4 pillows and a rolled-up towel underneath his head. BIPAP was applied at the start of the second study at an initial pressure of 8/4 cm H_2_O. The patient initially chose a medium size full-face mask but switched to a size large later on in the study due to mask leakage issues. The patient had difficulty tolerating BIPAP pressure of 20/16 cm H_2_O.

A dual mask was applied and titration started at pressure of 6/4 cm H_2_O with 2 liters/min of O_2_. At the pressure of 12/8 cm H_2_O with 3 liters/min of O_2_, most of abnormal respiratory events resolved. This pressure was easily tolerable, without any significant leakage (Tables 5 and 6). 

### 3.4. Case  4

This was a 68-year-old female with history of witnessed apneas and CHF. She was a chronic smoker. She was scheduled for a split night study and her AHI was 38 during the first part of sleep study with desaturations in low 70s. CPAP was applied around 2320. Pressure of 7 cm H_2_O was looking good until patient aroused. Subsequently, she started to have many central apneas; therefore, BIPAP was applied. At the BIPAP pressure of 14/10 cm H_2_O, 2 liters/min of O_2_ was added and it was increased to 3 liters/min at BIPAP pressure of 18/12 cm H_2_O. BIPAP and CPAP both failed and technician was unable to establish a final pressure due to numerous central apneas that were mainly during rapid eye movement sleep. She requested to end the study around 0200 because the mask was hurting her face. Sleep technician tried several different types of masks and a small Quattro full-face mask was used. She was a major mouth breather. The patient slept in the supine position for entire study.

In her second night of PSG, she was started on BIPAP titration with dual mask and 2 liters/min of O_2_. At the pressure of 14/10 cm H_2_O with 3 liters/min of O_2_, most apneas/hypopneas and snoring resolved (Tables 7 and 8).

## 4. Discussion

Clinicians are experiencing an ever increasing number of patients presenting with overlap syndromes to the surgical wards and operating rooms. The managements and monitoring of patients with overlap syndromes remain complicated. Both positive pressure ventilation and oxygen therapy [9] remain the standard of care for treatment of patients with overlap syndromes. However, poor mask fit due to facial and anatomical features reduces the efficacy of such therapy in the immediate postoperative period. Alternative strategies are needed to better provide oxygen and positive pressure ventilation. A new novel device described previously in the postoperative period [10] and now tested in our sleep laboratory may provide additional support to the surgical and medical providers.

Higher CPAP/BIPAP pressure can lead to higher leakage [11] and may increase nasal drying or congestion [12], cause pressure sores on the bridge of the nose, and contribute to difficulty in exhaling [13]. In current clinical practice, O_2_ is added to CPAP/BIPAP mask usually from a small hole into the mask or to CPAP/BIPAP hose; hence, added oxygen becomes diluted by CPAP/BIPAP flow and is also subject to leakage. Therefore, patients rarely get the benefit of supplemental O_2_. Adding O_2_ to the patient's mask is not practical because it can easily get disconnected when patient changes position during sleep. This is one of the most common patients' complaints when O_2_ line is directly connected to the mask. Connecting an adjustable cannula to full-face or nasal mask and delivering supplemental oxygen directly to nostrils rather than bleeding it into the mask space or hose will provide higher oxygen saturation during inhalation with the help of CPAP/BIPAP pressure.

As demonstrated in the four cases discussed previously, we were able to use the dual mask to improve respiratory parameters in all 4 patients with OSAS and overlap syndrome.

## 5. Conclusion

The goal is to deliver supplemental O_2_ directly to patients' nose in order to minimize dilution and leakage and to provide higher oxygenation with minimum CPAP/BIPAP pressure. That is expected to result in lower pressure-related side effects and subsequently higher compliance rates. Also delivering both room air and O_2_ via only one tube and one machine provides more convenience and decreases the chance of O_2_ disconnection by patient movements.

## Figures and Tables

**Table 1:** Treatment parameters and results.

Treatment	TIB	TST	Slp Eff	Apneas		Hypopneas		AHI	RDI	Ar + Aw		Min SpO2
RxI/RxE/RxO2	Minutes	Minutes	Percent	Count	Index	Count	Index	Index	Index	Count	Index	
8/4/0	22	6	27.3	1	10	0	0	10	10	3	30	83
9/5/0	22	14.5	65.9	3	12.4	7	29	41.4	41.4	12	49.7	86
10/6/2	17	13	76.5	4	3.2	9	41.5	60	60	11	50.8	88
11/7/2	44	38	86.4	2	3.2	1	1.6	4.7	4.7	2	3.2	86
12/8/2	28	15.5	55.4	0	0	0	0	0	0	12	46.5	87
14/10/2	52.5	27	51.4	8	17.8	1	2.2	20	20	24	53.3	84
16/12/2	47	43.5	92.6	0	0	0	0	0	0	4	5.5	89
13/9/2	26.5	1	3.8	0	0	0	0	0	0	1	60	89
18/14/2	14	14	100	5	21.4	1	4.3	25.7	25.7	0	0	90
19/15/2	28.5	28	98.2	4	8.6	0	0	8.6	8.6	1	2.1	93
20/16/2	25	19.5	78	2	6.2	0	0	6.2	6.2	6	18.5	92
21/17/2	60.5	56	92.6	3	3.2	1	1.1	4.3	4.3	9	9.6	90
23/19/3	6	3	50	0	0	0	0	0	0	2	40	92
22/18/3	32	30.5	95.3	0	0	0	0	0	0	2	3.9	91

TIB: time in bed.

TST: total sleep time.

Slp Eff: sleep efficiency.

AHI: apnea-hypopnea index.

RERA: respiratory effort-related arousal.

RDI: respiratory disturbance index. The respiratory disturbance index (RDI) is the total number of events (e.g., apneas, hypopneas, and RERAs) per hour of sleep. The RDI is generally larger than the AHI, because the RDI includes the frequency of RERAs, while the AHI does not.

Ar + Aw: arousal + awakening.

Ar + Aw Index: number of arousal and awakening per hour.

**Table 2:** Treatment parameters and results/with dual mask.

Treatment	TIB	TST	Slp Eff	Apneas		Hypopneas		AHI	RDI	Ar + Aw		Min SaO2
RxI/RxE/RxO2	Minutes	Minutes	Percent	Count	Index	Count	Index	Index	Index	Count	Index	
6/6/2	98	86	87.8	2	1.4	1	.7	2.1	2.1	7	4.9	92
8/8/2	178.5	150.5	84.3	0	0	3	1.2	1.2	1.2	52	20.7	94
10/10/2	127	98.5	77.6	0	0	2	1.2	1.2	1.2	10	6.1	95
0/0/0	.5	0	0	0	0	0	0	0	0	0	0	0
												—

**Table 3:** Treatment parameters and results.

Treatment	TIB	TST	Slp Eff	Apneas		Hypopneas		AHI	RDI	Ar + Aw		Min SpO2
RxI/RxE/RxO2	Minutes	Minutes	Percent	Ct	Index	Ct	Index	Index	Index	Ct	Index	
4/0/0	12	4.5	37.5	0	0	4	53.3	53.3	53.3	1	13.3	86
6/0/0	35.5	13.5	38	13	57.8	7	31.1	88.9	88.9	4	17.8	80
8/0/0	10	10	100	12	73.8	1	6	78	78	7	42	79
10/0/2	7	6.5	92.9	8	73.8	0	0	73.8	73.8	7	64.6	78
10/6/2	8.5	8.5	100	10	70.6	0	0	70.6	70.6	10	70.6	75
12/8/2	17	17	100	20	70.6	4	14.1	84.7	84.7	4	14.1	74
14/10/2	16	15.5	96.9	8	31	8	31	61.9	61.9	0	0	82
15/11/2	20	19	95	17	53.7	2	6.3	60	60	8	25.3	80

**Table 4:** Treatment parameters and results/with dual mask.

Treatment	TIB	TST	Slp Eff	Apneas		Hypopneas		AHI	RDI	Ar + Aw		Min SpO2
RxI/RxE/RxO2	Minutes	Minutes	Percent	Count	Index	Count	Index	Index	Index	Count	Index	
8/4/2	15.5	15.5	100	0	0	0	0	0	0	0	0	91
9/5/2	16.5	9.5	57.6	0	0	1	6.3	6.3	6.3	0	0	92
10/6/2	23	19.5	84.8	0	0	2	6.2	6.2	6.2	2	6.2	91
11/7/2	14.5	14.5	100	0	0	3	12.4	12.4	12.4	1	4.1	90
12/8/2	88.5	86.5	97.7	0	0	2	1.4	1.4	1.4	9	6.2	90
—	—	—	—	—	—	—						

**Table 5:** Treatment parameters and results.

Treatment	TIB	TST	Slp Eff	Apneas		Hypopneas		AH	RD	Ar + Aw		Min SpO2
RxI/RxE/RxO2	Minutes	Minutes	Percent	Count	Index	Count	Index	Index	Index	Count	Index	
8/4/0	15.5	15.5	100	1	3.9	4	15.5	19.4	19.4	0	0	81
10/6/2	17	17	100	1	13.3	4	14.1	17.6	17.6	0	0	76
12/8/2	11	11	100	1	5.5	8	43.6	49.1	49.1	4	21.8	78
14/10/2	31	31	100	1	1.9	4	7.7	9.7	9.7	0	0	79
16/12/2	24.5	24	98	1	2.5	3	7.5	10	10	2	5	79
18/14/2	14.5	14.5	100	3	12.4	5	20.7	33.1	33.1	5	20.7	70
20/16/3	23.5	4.5	19.1	1	13.3	1	13.3	26.7	26.7	3	40	71

**Table 6:** Treatment parameters and results/with dual mask.

Treatment	TIB	TST	Slp Eff	Apneas		Hypopneas		AHI	RDI	Ar + Aw		Min SpO2
RxI/RxE/RxO2	Minutes	Minutes	Percent	Count	Index	Count	Index	Index	Index	Count	Index	
6/4/2	51	3	5.9	0	0	5	100	100	100	1	20	89
8/6/2	117.5	83.5	71.1	2	1.4	9	6.5	7.9	7.9	34	24.4	87
10/8/3	21.5	19.5	90.7	0	0	0	0	0	0	10	30.8	92
12/8/3	96	86.5	90.1	0	0	2	1.4	1.4	1.4	9	6.2	92

**Table 7:** Treatment parameters and results.

Treatment	TIB	TST	Slp Eff	Apneas		Hypopneas		AHI	RDI	Ar + Aw		Min SpO2
RxI/RxE/RxO2	Minutes	Minutes	Percent	Count	Index	Count	Index	Index	Index	Count	Index	
4/0/0	12.5	8.5	68	4	28.2	7	49.4	77.6	77.6	4	28.2	70
6/0/0	15.5	15.5	100	4	15.5	2	7.7	23.2	23.2	2	7.7	75
7/0/0	23	23	100	1	20	0	0	2.6	2.6	0	0	78
8/0/0	12	6	50	2	20	2	20	40	40	0	0	80
9/0/0	9	9	100	13	86.7	1	6.7	93.3	93.3	7	46.7	83
10/6/0	18	18	100	23	76.7	3	10	86.7	86.7	22	73.3	81
12/8/0	15	14	93.3	22	94.3	1	4.3	98.6	98.6	12	51.4	80
14/10/2	8.5	8.5	100	11	77.6	3	21.2	98.8	98.8	3	21.2	85
16/12/2	14.5	14.5	100	19	78.6	1	4.1	82.8	82.8	4	16.6	80
18/12/3	33	26	78.8	19	43.8	4	9.2	53.1	53.1	5	11.5	86

**Table 8:** Treatment parameters and results/with dual mask.

Treatment	TIB	TST	Slp Eff	Apneas		Hypopneas		AHI	RDI	Ar + Aw		Min SaO2
RxI/RxE/RxO2	Minutes	Minutes	Percent	Count	Index	Count	Index	Index	Index	Count	Index	
8/4/2	36	35.5	98.6	2	3.4	5	8.5	11.8	11.8	6	10.1	92
9/6/2	17	17	100	0	0	1	3.5	3.5	3.5	7	24.7	85
10/6/2	17	16.5	97.1	2	7.3	1	3.6	10.9	10.9	3	10.9	88
11/6/2	12.5	12.5	100	1	4.8	6	28.8	33.6	33.6	3	14.4	77
12/8/2	28	13.5	48.2	0	0	2	8.9	8.9	8.9	3	13.3	88
13/8/2	24	16	66.7	3	11.3	0	0	11.3	11.3	8	30	94
14/8/2	9.5	9.5	100	2	12.6	2	12.6	25.3	25.3	3	18.9	95
14/9/2	19	19	100	0	0	0	0	0	0	3	9.5	93
14/10/3	42.5	41.5	97.6	1	1.4	2	2.9	4.3	4.3	5	7.2	95
15/10/3	30.5	27.5	90.2	0	0	4	8.7	8.7	8.7	1	2.2	87
16/10/3	15.5	15.5	100	0	0	4	15.5	15.5	15.5	2	7.7	85
14/10/3	48	47.5	99	0	0	3	3.8	3.8	3.8	3	3.8	90
15/10/3	46	25	54.3	0	0	0	0	0	0	1	2.4	93
16/12/3	31.5	25	79.4	1	2.4	1	2.4	4.8	4.8	5	12	92
